# Editorial: Imaging and Mechanism of Leukocyte Recruitment and Function in Inflammation and Infections

**DOI:** 10.3389/fcell.2021.690003

**Published:** 2021-05-07

**Authors:** Hao Sun, Yuqing Huo, Zhichao Fan

**Affiliations:** ^1^Department of Medicine, University of California, San Diego, La Jolla, CA, United States; ^2^Department of Cellular Biology and Anatomy, Vascular Biology Center, Medical College of Georgia, Augusta University, Augusta, GA, United States; ^3^Department of Immunology, School of Medicine, UConn Health, Farmington, CT, United States

**Keywords:** leukocytes, leukocyte adhesion, leukocyte recruitment, leukocyte imaging, intravital imaging

The recruitment of leukocytes plays essential roles during protective and pathological immune responses of many diseases. Recruitment from circulation involves several processes such as rolling and adhesion to endothelial cells lining blood vessel inner walls, transmigration across vessel inner walls into surrounding tissue (diapedesis), and migration into other tissues. Leukocyte recruitment is precisely regulated by a complex network of adhesion molecules, including integrins, and their ligands to ensure the proper positioning of immune cells in local microenvironments. Many types of leukocytes help regulate immune defense and inflammation, including T cells, B cells, and natural killer cells, as well as myeloid cells, such as neutrophils, monocytes, macrophages, and dendritic cells (DCs). Microscopic imaging is a powerful visualization method to investigate leukocyte recruitment and functions.

The Frontiers Research Topic “Imaging and Mechanism of Leukocyte Recruitment and Function in Inflammation and Infections” highlights 31 recent studies that use imaging and other techniques to investigate leukocyte recruitment and functions in different infectious or non-infectious diseases.

## General Mechanisms of Leukocyte Recruitment

Leukocytes can dynamically increase the affinity of integrins for their ligands (activation), an event central to many of their functions. Leukocyte integrins are critical for innate and adaptive immune responses and contribute to many immune-related conditions. It is not surprising then that integrins have emerged as promising treatment targets for autoimmune diseases and cancer. In a comprehensive overview, Park et al. detailed the role of integrin interactions in both health and disease. This review highlighted irisin, a novel exercise-dependent secretory integrin ligand, and its potential regulatory functions, proposing the αV integrin as a potential receptor for irisin, which is based on most of the recent publications about integrins and integrin-irisin interactions. Leukocytes homing from blood to gut-associated lymphoid tissue (GALT) are mediated by the interaction between integrin α4β7 with its ligand mucosal vascular addressin cell adhesion molecule 1 (MAdCAM-1). In an original research article, Yuan et al. demonstrated that heparan sulfate (HS), which is an acidic linear polysaccharide with a highly variable structure, could promote integrin α4β7-mediated cell adhesion on MAdCAM-1 under shear flow by imitating the negative charges of the extracellular microenvironment, suggesting the negative charges facilitate α4β7-MAdCAM-1 mediated leukocyte migration to GALT.

Chemokines guide leukocyte homing and trafficking through G protein-coupled receptor (GPCR) signaling pathways. C-C chemokine receptor-like 2 (CCRL2) shares structural and functional similarities with atypical chemokine receptors (ACKRs), which is a subset of chemokine receptors unable to activate signal transduction through G proteins. Schioppa et al. provided a valuable general overview of the current understanding of CCRL2 in terms of expression regulation, ligand binding, as well as its functions in leukocyte migration, which may provide novel therapeutic strategies for the control of inflammation and tumor immune surveillance. Along the same line, a recent study by Kashem et al. evaluated the effect of Toll-like interleukin-1 receptor regulator (TILRR)-induced pro-inflammatory cytokines/chemokines on the migration of immune cells. TILRR has been identified as an important modulator of inflammation responsive genes. This work demonstrated that culture supernatants from TILRR-overexpressed cervical epithelial cells can increase the migration of THP-1 monocytes and MOLT-4 T-lymphocytes, which may influence immune cell infiltration in tissues.

Leukocyte recruitment is regulated by the interaction between endothelium-expressed proteins and circulating leukocytes. The involvement of glycan and glycan-binding proteins in the leukocyte recruitment cascade has been well-studied. A review by Krautter and Iqbal focused on the role of glycan-glycan binding protein interactions in the regulation of leukocyte trafficking during inflammation. Given the prevalence and importance of glycan-mediated interactions for the regulation of leukocyte function, this review also highlighted the therapeutic targeting of glycans and glycan-binding proteins in the context of leukocyte recruitment during inflammation.

Palmitoylation is the covalent attachment of the fatty acid palmitic acid to cysteine. Recently, many studies have shown that leukocyte proteins undergo palmitoylation, including adhesion molecules, cytokine/chemokine receptors, T cell co-receptors, and signaling factors. In a review focusing on the function of palmitoylated proteins in leukocytes, Yang et al. illustrated the central role of palmitoylation in leukocyte migration and function. They proposed targeting palmitoylation may serve as a promising therapeutic strategy for aberrant immune responses.

Phosphoinositides are a family of minority acidic phospholipids in cell membranes with numerous functions, including attracting phospholipase C, protein kinase C, proteins involved in membrane budding and fusion, proteins regulating the actin cytoskeleton, and others. A review from Montaño-Rendón et al. summarized the most recent findings concerning the metabolic regulation of phosphoinositides during the leukocyte lifecycle. They mainly focused their attention on cellular processes, including diapedesis, migration, and phagocytosis, highlighting the importance of proper phosphoinositide interconversion to maintain cellular homeostatic functions.

The activation of complement receptors helps to regulate inflammation, leukocyte extravasation, and phagocytosis; activation also contributes to the adaptive immune response. This particular topic is illustrated in the review article by Vandendriessche et al., which focused on complement receptors and their roles in regulating phagocytosis and leukocyte recruitment during immunity and inflammation. Complement receptors participate in firm endothelial adhesion, diapedesis, and leukocyte chemotaxis toward the inflammatory trigger. Within tissues, they are involved in phagocytosis and cell type-specific immune regulatory roles. Chemotaxis and phagocytosis are fundamental processes that protect the body and maintain tissue homeostasis. Thus, the complement system is a promising therapeutic target for many diseases.

The phosphodiesterases (PDEs) that degrade cyclic adenosine monophosphate (cAMP) and cyclic guanosine monophosphate (cGMP) have been studied for over 40 years. Several drugs have been developed that target cGMP signaling, while drugs targeting cAMP have not been that successful; this is partly because cAMP is ubiquitous and critically required in virtually all tissues, while cGMP is more prominent and confined to cardiovascular tissues. cAMP-specific PDE4 and PDE8 are higher potential targets for inflammatory diseases. Epstein et al. provided a comprehensive overview of the potential unique roles of PDE8 (PDE8A) in T cell motility and T cell-mediated inflammation, with several details on the distinct signaling properties of PDE8 in a huge variety of *in vivo* and *ex vivo* models, and in different cell types. This provided evidence for the existence of a unique PDE8-dependent signaling complex disruptor.

Recently, much attention has been drawn to the leukocyte recruitment role of extracellular RNA (exRNA), which is released from cells under conditions of injury or vascular disease. Preissne et al. highlighted articles that investigated the role of the exRNA on various steps of leukocyte recruitment within the innate immune response. Based on their work in a variety of preclinical animal models, exRNA constituted a versatile damaging factor in several inflammatory cardiovascular diseases, whereby the natural endonuclease RNase1 provided an effective and safe antagonist by inhibiting or preventing the pathological effects of exRNA—suggesting it may serve as a new therapeutic target.

Neutrophils are a marker of innate immune activation against invading microorganisms, as they are the first leukocytes to migrate from the circulation to inflamed or infected sites. The research article by Liu et al. described the mechanisms of endothelium-neutrophil interactions on bactericidal activity. They disclosed that interleukin (IL)-1α promoted neutrophil adhesion by upregulating cell adhesion molecules (CAMs) during the early phase, while oxidative phosphorylation induced transmigration of neutrophils against bacteria. Understanding the role of neutrophils during neuroinflammation is of great importance, and the observations by Kim et al. on the migration of microglia and neutrophils in the inflamed brain are very intriguing. Using two-photon intravital microscopy, the authors found that neuroinflammation leads to both influx and transendothelial migration of neutrophils in the brain and promotes their interaction with microglia. These observations may indicate that neutrophils can prime microglia to initiate a neuroinflammatory response and deliver pro-inflammatory signals to the periphery through reverse trans-endothelial migration (rTEM).

Barcellos et al. discussed interesting reports that focused on how physical training regulates exercise-induced inflammation and performance. This study demonstrated that acute high-intensity exercise promoted inflammation, including an increase of IL-6 in blood, enhanced rolling and adhesion of leukocytes (primarily neutrophils) by intravital imaging, and promoted neutrophil chemotaxis *in vitro*. Aerobic training diminished exercise-induced inflammation in skeletal muscle tissue. They also proposed that reactive oxygen species (ROS) are potential factors affecting aerobic performance and inflammatory response.

In a review, Schwartz et al. focused on an aspect of the leukocyte trans-endothelial migration (or diapedesis), which is an important process that allows leukocytes to reach the sites of tissue damage or infection to elicit an adequate response linked to innate or acquired immunity and inflammation. This review presented the biomechanical mechanisms involved in trans-endothelial crossing of leukocytes and summarized the various imaging techniques currently used with their advantages and limitations.

DCs are powerful antigen-presenting cells, and their migration ability is the key to initiating protective pro-inflammatory and tolerant immune responses. Recent studies have emphasized the importance of DC migration in maintaining immune surveillance and tissue homeostasis, as well as the pathogenesis of a series of diseases. Feng et al. provided a comprehensive view of the migration of DCs, especially under various environments, which may help to develop new therapeutic and vaccination strategies for diseases. Along the same line, Lin et al. demonstrated that sCD83 could inhibit DC-T synapse formation by decreasing Rab1a activation to disrupt F-actin and MHC-II accumulation at sites of DC-T contact, providing a possible mechanism for the role of DCs in immunological synapse formation. In experimental autoimmune uveitis (EAU) mice, sCD83-treated DCs decreased the number of T cells in the eyes and lymph nodes, and alleviated symptoms of EAU.

Monocytes (Mo) and macrophages (Mφ) are key components of the innate immune system and actively participate in the development of many autoimmune diseases. The infiltration of Mo and Mφ in diseased tissues is a hallmark of several autoimmune diseases. In this topic, Yang et al. investigated the role played by adenosine monophosphate-activated protein kinase (AMPK) signaling-dependent metabolic reprogramming in Mo/Mφ migration and survival in metabolic diseases, such as high-fat diet (HFD)-induced diabetes and atherosclerosis. They observed enhanced protein kinase adenosine monophosphate-activated catalytic subunit alpha 1 (PRKAA1/2) expression in adipocyte-associated-leukocytes in mice on a HFD, and highlighted a critical role of PRKAA1 in Mo and Mφ metabolic modulation, migration, and survival.

An original article from Tan et al. showed the induction of inflammation in alveolar cells by the interaction of triggering receptor expressed on myeloid cells-1 (TREM-1) with extracellular cold-inducible RNA-binding protein (CIRP). The authors showed that adding recombinant eCIRP increases TREM-1 expression in alveolar epithelial cells and induces production of cytokines IL-6 and CXCL2. This study provides insights into molecular mechanisms of acute lung injury, which is a life-threatening condition involved in many diseases, with additional importance during the COVID-19 pandemic.

Phagocytosis is an essential response of innate immune cells that involes recognition, engulfment, and degradation of foreign or dead material. Westman and Grinstein provided a comprehensive view of how phagosomal pH is regulated during phagocytosis, including why it varies among different professional phagocytes, and how host defense and pathogen escape occur in phagocytosis. Indeed, metabolite transport and utilization are both exquisitely pH-sensitive events. Therefore, the establishment and regulation of luminal pH should be the core content of host-pathogen interactions in the future.

## Leukocyte Imaging

With the advent of new imaging techniques, new ways of visualizing immune cells have emerged in recent years, enabling more detailed discoveries about cell properties, functions, and interactions. Jorch and Deppermann wrote a comprehensive review on novel imaging techniques that provide insights into neutrophil and macrophage functions under physiological and pathological conditions. This article introduced several optical imaging techniques, including epifluorescent microscopy, confocal microscopy, and multi-photon microscopy. They also discussed light-sheet microscopy used after optical tissue clearing, which is valuable to visualize the structure and distribution of neutrophils and macrophages in fixed tissue.

The use of intravital microscopy over recent decades significantly improved our understanding of neutrophil trafficking and functions in various microenvironmental niches under homeostasis and in response to infections. Such technological advances allow studies on the temporal and spatial regulation of neutrophil functions, as well as the emerging role of tissue-resident neutrophils in the lung, skin, spleen, and lymph nodes. Through the perspective of intravital microscopy, De Filippo and Rankin presented recent studies on the function, migratory behavior, and cell-cell interactions of neutrophils in various tissues, outlining the importance of neutrophil subsets, their functions under homeostasis, and their responses to infection. They also commented on how understanding these processes in greater detail at a molecular level can lead to new therapeutics.

The lung is one of the most difficult organs to assess using intravital optical imaging due to its enclosed position within the body, delicate nature, and vital role in sustaining proper physiology. Alizadeh-Tabrizi et al. reviewed studies of lung intravital imaging and lung disease mechanisms. They introduced the microscopy modalities used for intravital imaging and the procedure for lung intravital microscopy in mice. They also commented on infection and inflammation models of lung diseases in which lung intravital imaging was used, and listed a number of limitations of the technique.

Margraf and Sperandio provided a “practical guide” for intravital observation of leukocyte trafficking and homeostasis in the developing mouse fetus to further understand immune defense and hemostatic capability. They provided a methodology for visualizing blood plasma and vessel walls, as well as cells in circulation. This model offers a potential technique to study *in vivo* processes in the developing mouse fetus.

By combining optical clearing and multi-photon microscopy, Szabo-Pardi et al. investigated the recruitment of peripheral leukocytes to key tissues of the pain system, the dorsal root ganglia (DRG) and sciatic nerve, after nerve injury. They observed robust sexual dimorphism in leukocyte recruitment to lumbar DRGs after nerve injury. As mentioned above, intravital imaging was also used to study leukocytes in the brain (Kim et al.) and muscles (Liu et al.).

Arthritis, an immune-mediated inflammatory disease, is induced by the inappropriate accumulation and activation of leukocytes. In a comprehensive review, Manning et al. provided an in-depth synopsis of rheumatic joint inflammatory imaging describing the evolving techniques to visualize migrating leukocytes. It elegantly described the inflammatory processes and the evolution of imaging methods along with the leukocyte profile of the joint and what is learned as the techniques advance. It showed the development and improvement of different techniques, which can improve our knowledge of this disease and allow observers to track treatment responses.

Super-resolution microscopy is a powerful tool for studying molecular details in leukocytes. An original article from Joly et al. explored whether endosomes contribute to NADPH oxidase 2 (NOX2) delivery to the phagosome in neutrophils and found that a fraction of the early and recycling endosomes contained NOX2. They used super-resolution microscopy for the first time to observe NOX2 organization in the membrane of PLB-985 cells. They observed a rise in the number of nanoclusters during phagocytosis that required the presence of the cytosolic oxidase subunit p47phox.

## Leukocyte Recruitment and Function in Diseases

Recent studies have highlighted the role of inflammation in obesity, diabetes, hypertension, autoimmune disease, and cardiovascular diseases, including stroke and atherosclerosis. Atherosclerosis is a chronic inflammatory disease that occurs within the arterial wall and is initiated mainly in response to endogenous ligands, particularly oxidized lipoproteins, which stimulate both innate and adaptive immune responses. The innate response starts with the activation of endothelial cells in vessel walls and Mo/Mϕ activation. It is rapidly followed by an adaptive immune response to an array of potential antigens presented to effector T lymphocytes by antigen-presenting cells, such as DCs. Thus, leukocyte adhesion and recruitment to the vascular intima is a key event in early atherosclerosis.

Marchini et al. provided a comprehensive overview of the mechanisms underlying leukocyte recruitment in the setting of cardiovascular disorders, including myocardial infarction and atherosclerosis. They described the inhibition of receptors and ligands involved in the generation, adhesion, and transmigration of leukocytes that revealed a great potential for anti-leukocyte therapies at the preclinical stage for cardiovascular diseases.

Type 2 diabetes is a metabolic disorder. However, recent research suggests that type 2 diabetes is also an autoimmune disease. Pezhman et al. provided a valuable general overview of the dysregulation of leukocyte trafficking in type 2 diabetes in recent decades, including the changes in expression of adhesion molecules, chemokines, and chemokine receptors, innate and adaptive immune cells, and the subsequent immune response. They also discussed the potential therapeutic avenues targeting leukocyte trafficking.

A key element in the pathogenesis of inflammatory bowel disease (IBD) is a massive influx of immune cells into the gastrointestinal mucosa. Aberrant infiltration of mononuclear phagocytes, neutrophils, and inflammatory lymphocytes is observed in the colonic lamina propria of IBD patients. Leukocyte trafficking is, therefore, a key determinant of the immune responses that take place in the gut. Thus, this rapid leukocyte recruitment from the circulation into GI during IBD provides a potential target for pharmaceutical inhibition of the inflammation. Luzentales-Simpson et al. reviewed multiple proposed mechanisms of action for the integrin α4β7 blocking antibody vedolizumab used to treat IBD. Understanding the mechanism of action of this drug will allow a determination of the primary mechanisms of disease treatment in people with IBD. They also described the potential effects of vedolizumab on innate immune cells (macrophages, monocytes, and DCs) in particular.

In the past few years, miR-138 has been identified as a putative tumor suppressor and is found down-regulated in most human cancer types. Multiple targets of miR-138 have been identified previously, including key molecules involved in proliferation, apoptosis, invasion, and migration. The review by Song et al. investigated the immune-regulatory mechanisms of miR-138-5p in the non-small-cell lung cancer (NSCLC) microenvironment and tumor proliferation. They revealed that miR-138 has two essential functions in NSCLC therapy by targeting PD-L1/PD-1: regulating the immune response in the tumor micro-environment; and inhibiting proliferation of NSCLC cells by decreasing expression of Cyclin D3, Ki67, and minichromosome maintenance. This comprehensive review provided depth in understanding the molecular mechanism of miR-138 as a tumor suppressor in NSCLC, which is critical in therapeutic applications.

Stroke is currently the third-leading cause of death in the United States. During ischemic stroke, the blood-brain barrier is significantly damaged, resulting in the infiltration of peripheral immune cells and dramatic changes in cytokine levels. Bernstein and Rom studied the impacts of let-7 microRNA families on cytokine release and neurovascular space following ischemic stroke in mice. Using *in vitro* luciferase reporter assays, they further identified C-X-C motif chemokine ligand 1 (human as IL-8) as a potential target of let-7 g, and interferon gamma-induced protein-10 as a target of miR-98, respectively. They proposed that let-7 microRNAs may inhibit the release of cytokines at the blood-brain barrier and thus regulate endothelial-immune reactions following ischemic stroke.

In conclusion, this Research Topic provides detailed regulatory roles and molecular mechanisms for understanding leukocyte recruitment in health and disease. We would like to thank all the authors and referees for their valuable contributions. We believe that the collection of articles included in the topic will be of interest to all researchers studying molecular and cellular mechanisms of leukocyte migration and homing in development and diseases, enabling them to appreciate how a clearer understanding of these mechanisms can inspire therapeutics and diagnostics.

## Author Contributions

HS and ZF conceived the idea, designed, and edited the manuscript. YH corrected the manuscript. All authors listed have approved the work for publication.

## Conflict of Interest

The authors declare that the research was conducted in the absence of any commercial or financial relationships that could be construed as a potential conflict of interest.

